# Supplementary figures and images for: Exercise promotes satellite cell contribution to myofibers in a load-dependent manner
Source: Skelet Muscle. 2020 Jul 9;10:21. doi: 10.1186/s13395-020-00237-2 (PMC7346400; doi:10.1186/s13395-020-00237-2)

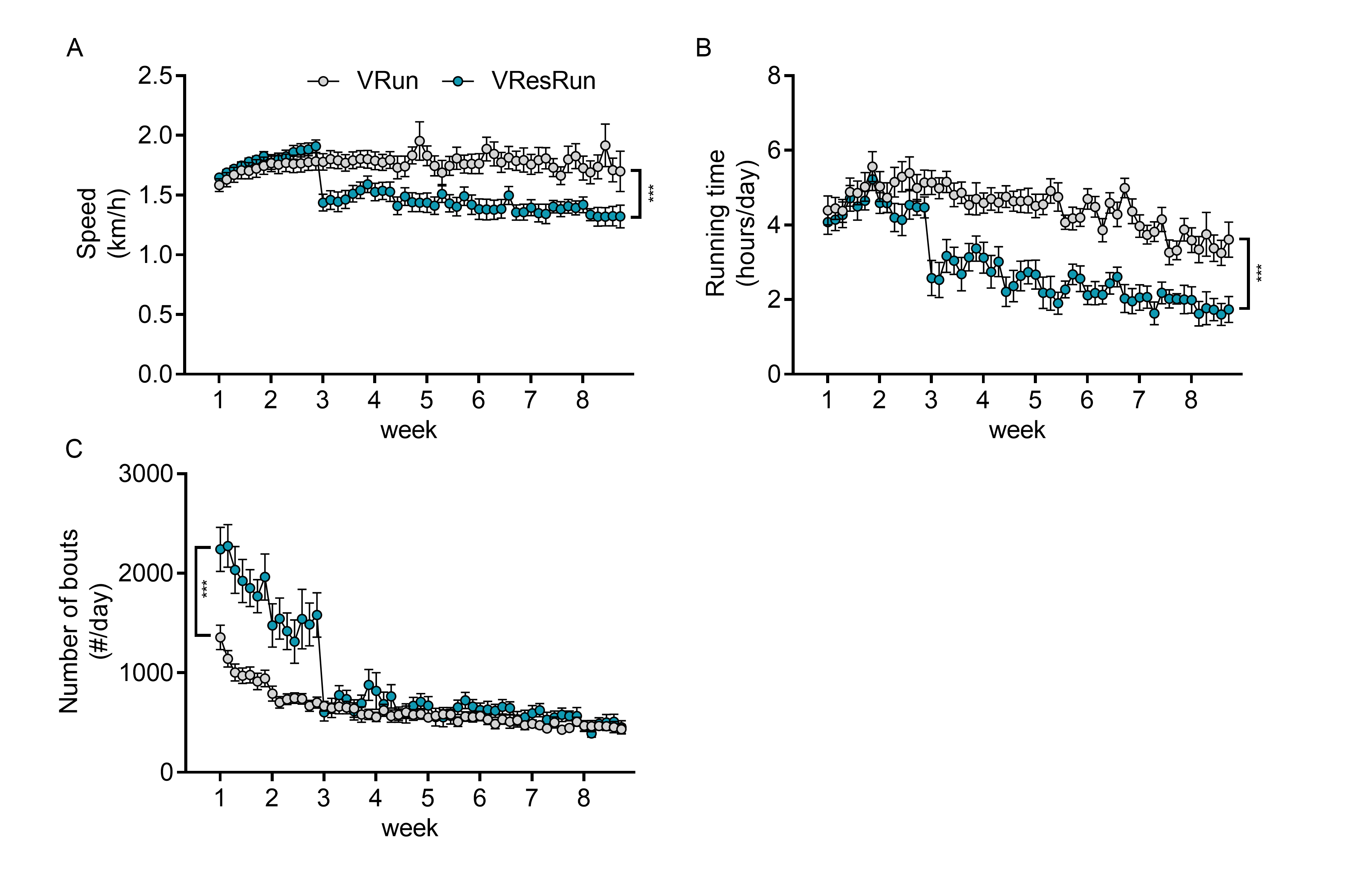

Supplement: Supplementary file 2 — Additional file 1: Figure S1. Running pattern. (A) Average running speed, (B) running time and (C) number of bouts per night throughout the exercise protocol (n = 16 mice per group). Statistics: two-way ANOVA test with a Bonferroni post hoc test. Group x time interaction is indicated in the figure. (*p <0.05; **p < 0.01; ***p < 0.001). Line graphs represent mean ± SEM (error bars). [file 13395_2020_237_MOESM1_ESM.tif]

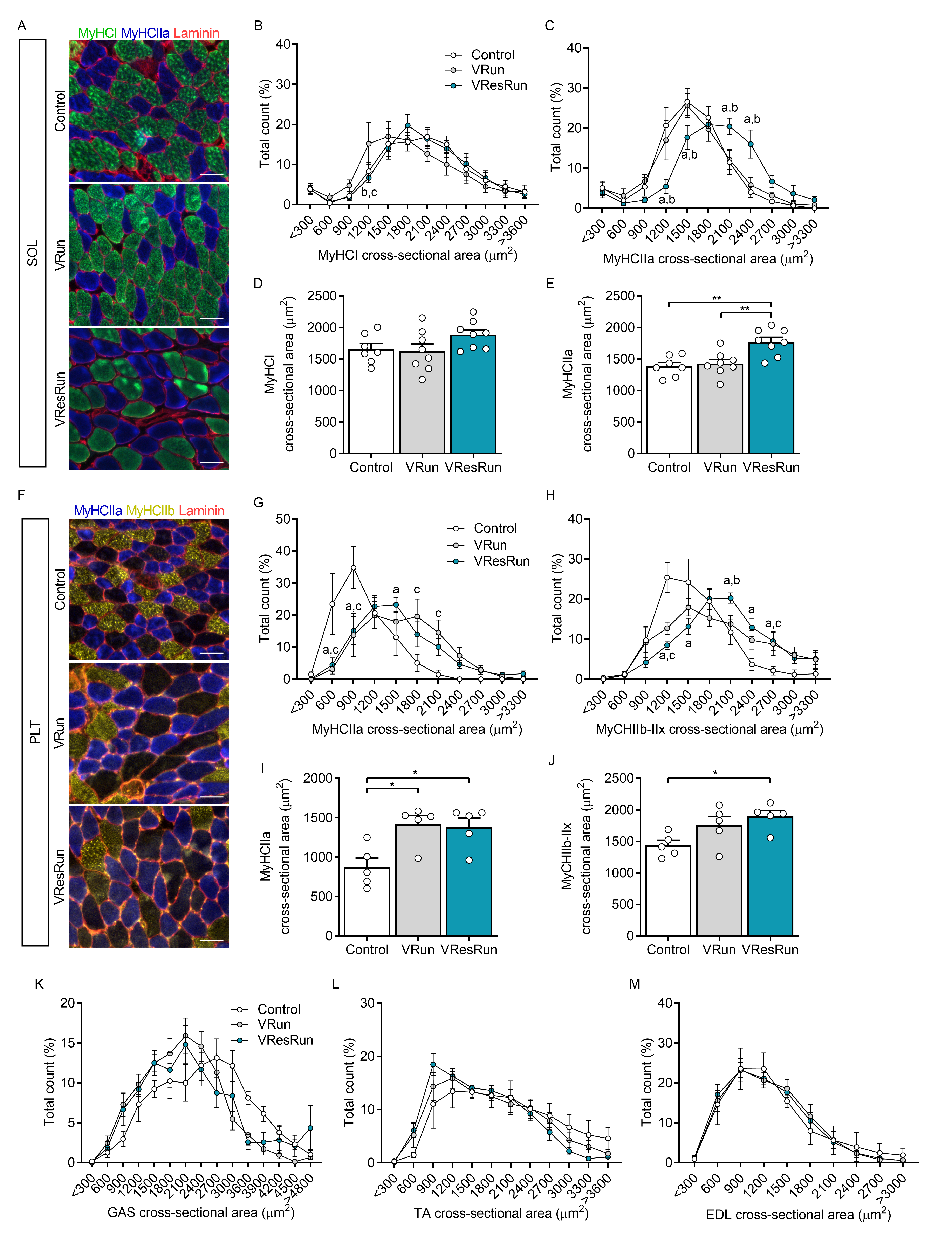

Supplement: Supplementary file 3 — Additional file 2: Figure S2. Muscle hypertrophy is muscle dependent, fiber type dependent and load-dependent. (A) Representative cross-sections for m. soleus (SOL) stained for MyHCI (green), MyHCIIa (blue) and Laminin (red). (B,C) Quantification of fiber cross-sectional area distribution and (D,E) average fiber cross-sectional area. (F) Representative cross-sections for plantaris (PLT) stained for MyHCIIa (blue), MyHCIIb (yellow) and laminin. (G,H) quantification of fiber cross-sectional area distribution and (I,J) average fiber cross-sectional area. (K) Fiber cross-sectional area distribution for gastrocnemius (GAS), (L) tibialis anterior (TA) and (M) extensor digitorum longus (EDL). Statistics: one-way ANOVA test with Tukey correction for multiple comparisons (*p < 0.05; **p < 0.01; ***p < 0.001) (a, p < 0.05 VResRun compared to Control; b, p < 0.05 VResRun compared to VRun; c, p < 0.05 VRun compared to Control). Bar graphs and line graphs represent mean ± SEM (error bars). Scale bar, 100 μm. [file 13395_2020_237_MOESM2_ESM.tif]

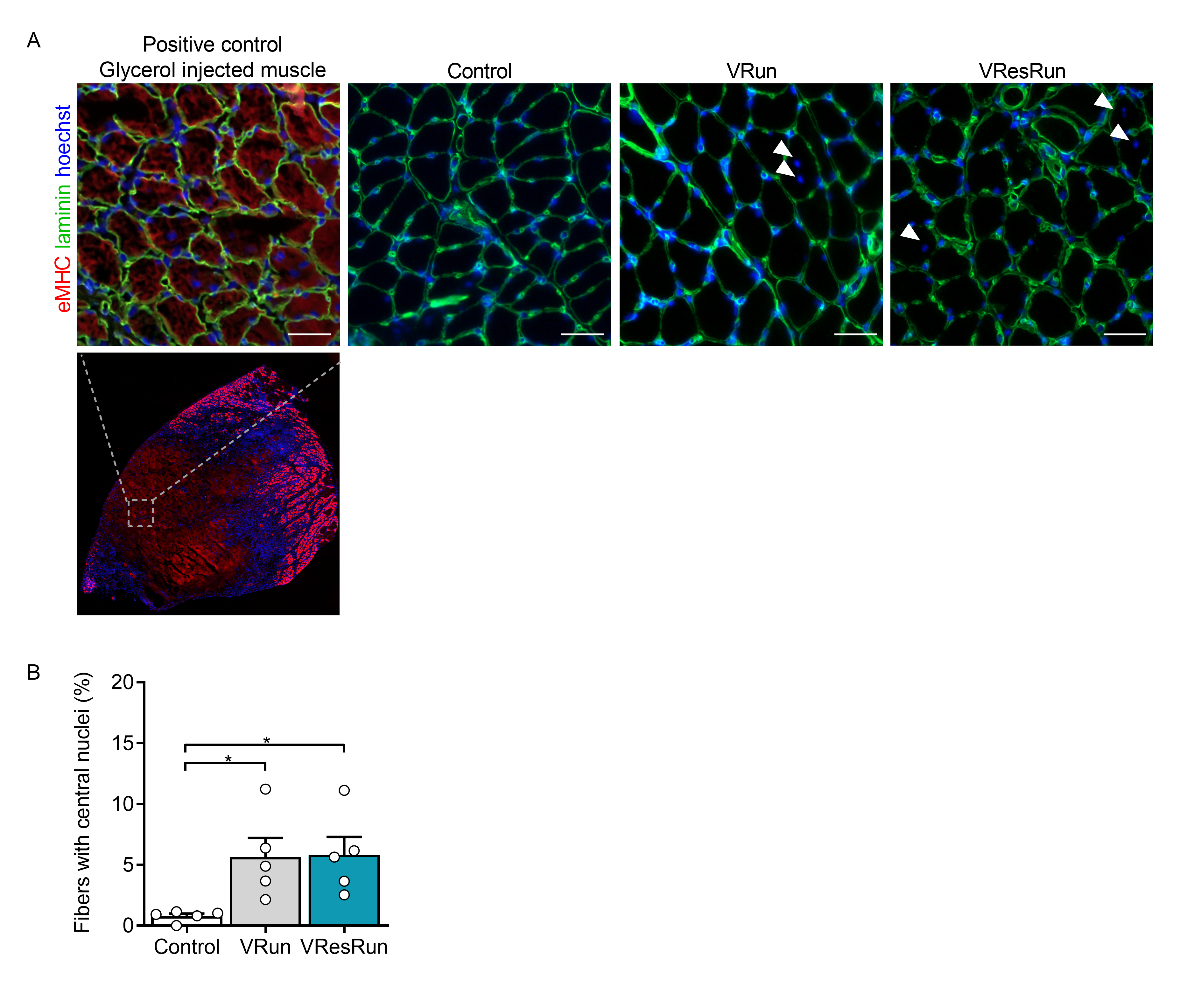

Supplement: Supplementary file 4 — Additional file 3: Figure S3. Fiber remodeling and central nuclei. (A) Myofibers stained for embryonic myosin heavy chain (eMHC), laminin and hoechst. No eMHC+ fibers were detected in Control, VRun, VResRun. Glycerol injected muscle was used as a positive control. (B) Quantification of fibers containing one or more centrally located nucleus. Arrows indicate central nuclei. Bar graph represents mean ± SEM (error bars). Statistics: one-way ANOVA test with Tukey correction for multiple comparisons (*p < 0.05). Each dot represents a single mouse. Scale bar, 50 μm. [file 13395_2020_237_MOESM3_ESM.tif]

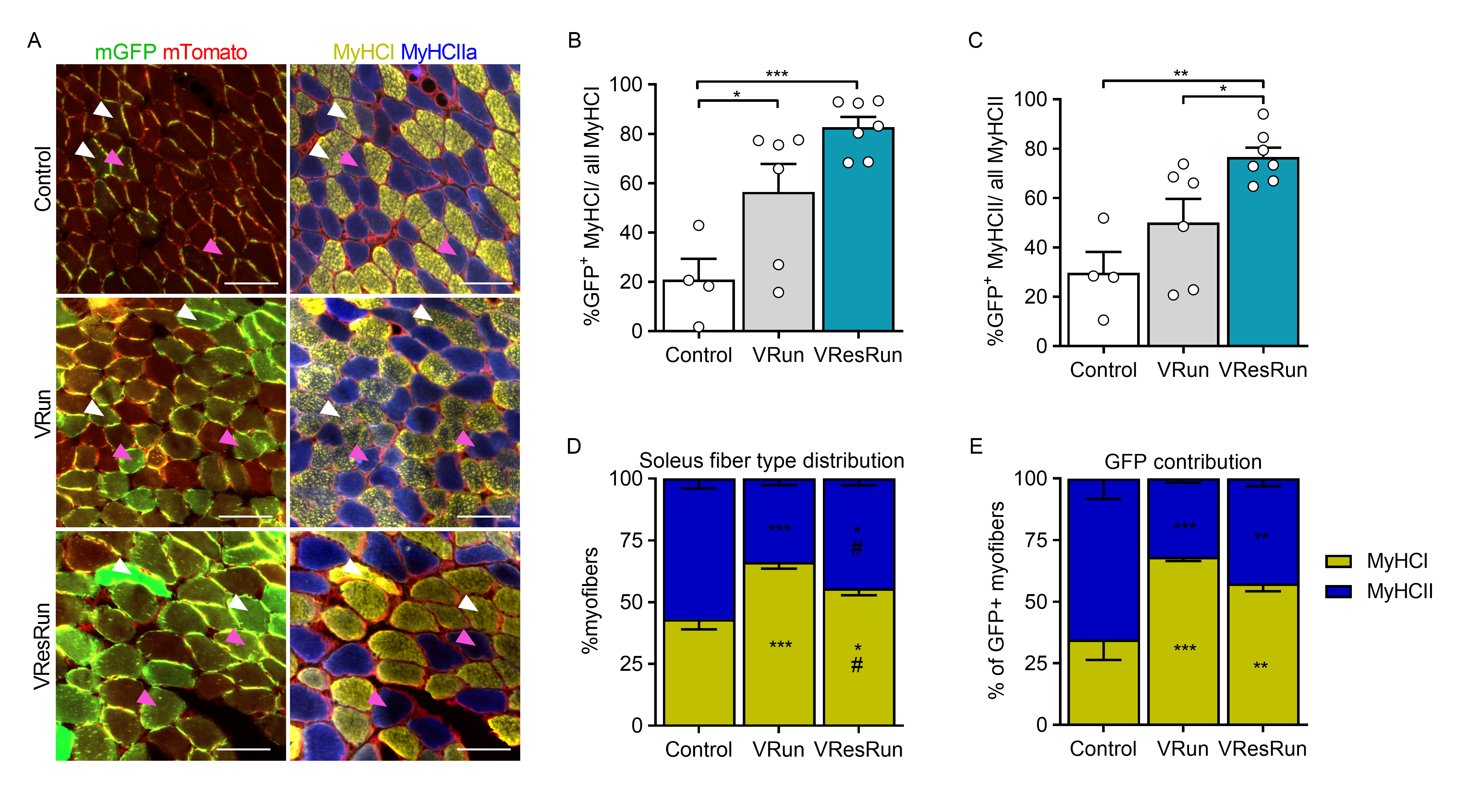

Supplement: Supplementary file 5 — Additional file 4: Figure S4. SC fusion is not fiber type dependent. (A) Representative sequential cross-sections for soleus for mGFP and mTomato (left panel) and stained for MyHCI (yellow) and MyHCIIa (blue) (right panel). (B) Quantification of GFP+ fibers for MyHCI and (C) MyHCIIa. (D) Fiber type distribution and (E) fiber type distribution of GFP+ fibers. Statistics: one-way ANOVA test with Tukey correction for multiple comparisons (*p < 0.05; **p < 0.01; ***p < 0.001). Each dot represents a single mouse. Bar graphs and line graphs represent mean ± SEM (error bars). White arrows indicate GFP+ MyHCI+ fibers, purple arrows indicate GFP+ MyHCIIa+ fibers. Scale bar, 100 μm. [file 13395_2020_237_MOESM4_ESM.tif]

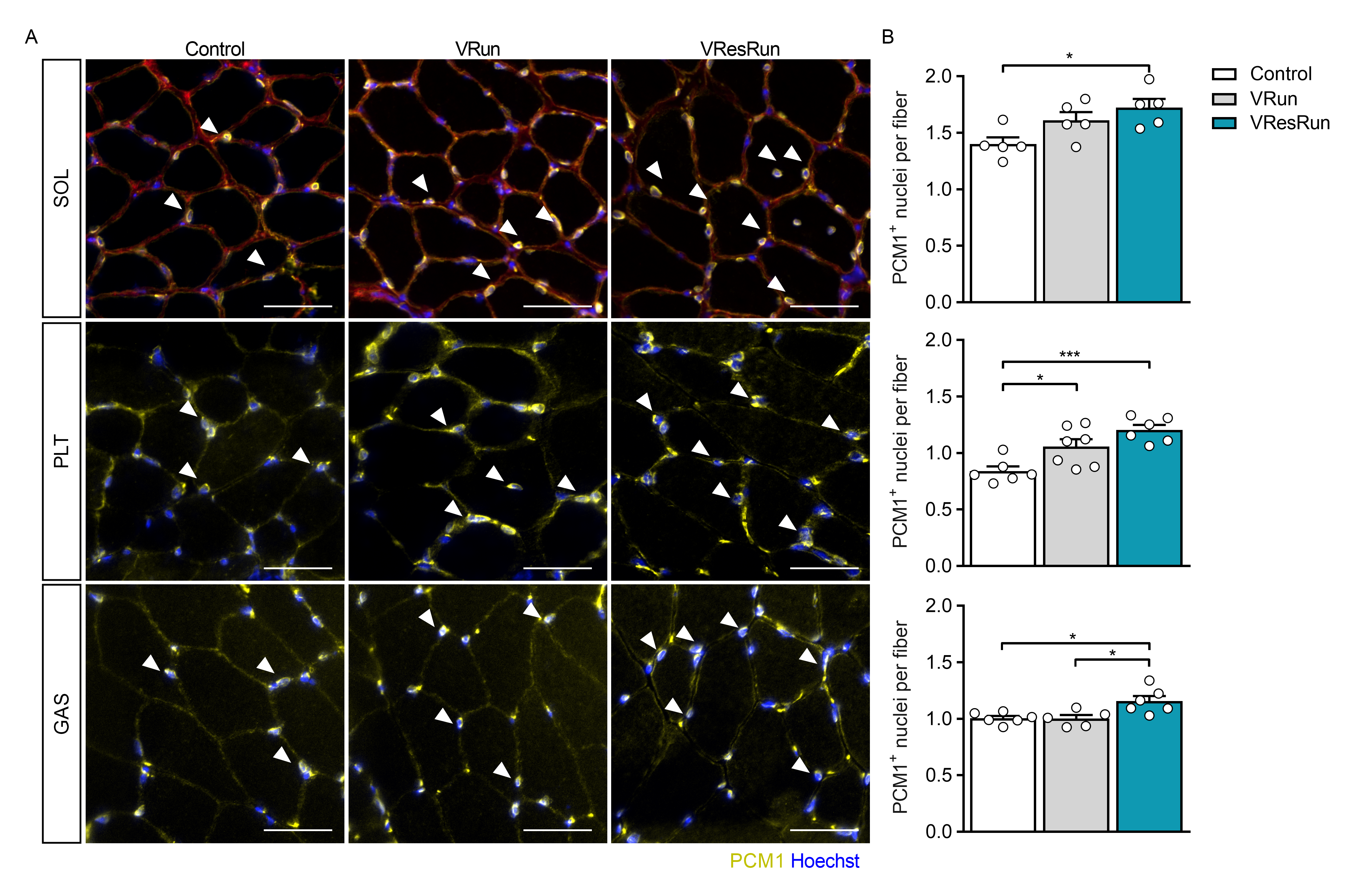

Supplement: Supplementary file 6 — Additional file 5: Figure S5. Myonuclear accretion is load-dependent. (A) Representative cross-sections of soleus, plantaris and gastrocnemius stained for PCM1 (yellow) and Hoechst (blue). (B) Quantification of PCM1+/Hoechst+ nuclei per fiber. Statistics: one-way ANOVA test with Tukey correction for multiple comparisons (*p < 0.05; **p < 0.01; ***p < 0.001). Bar graphs and line graphs represent mean ± SEM (error bars). White arrows indicate PCM1+ nuclei. Scale bar, 50 μm. [file 13395_2020_237_MOESM5_ESM.tif]
