# Supplementary material for: Exercise promotes satellite cell contribution to myofibers in a load-dependent manner
Source: Skelet Muscle. 2020 Jul 9;10:21. doi: 10.1186/s13395-020-00237-2 (PMC7346400; doi:10.1186/s13395-020-00237-2)
Supplement: Supplementary file 7 — Additional file 7: Table S1. Phenotypic characterization and muscle weights normalized to tibia length. Statistics: one-way ANOVA test with Tukey correction for multiple comparisons (*p < 0.05 VResRun vs. Control). Values represent mean ± SEM. n = 9-12 mice per group. [file 13395_2020_237_MOESM7_ESM.docx]

**Table S1**

|  | Control | VRun | VResRun |
| --- | --- | --- | --- |
| **Whole muscle weights** |  |  |  |
| SOL (mg)/tibia length (cm) | 5.15. ± 0.28 | 5.38 ± 0.21 | 6.39 ± 0.36* |
| PLT (mg)/tibia length (cm) | 7.10 ± 0.39 | 7.39 ± 0.21 | 7.13 ± 0.44 |
| GAS (mg)/tibia length (cm) | 58.81 ± 3.50 | 58.74 ± 1.09 | 56.70 ± 1.65 |
| TA (mg)/tibia length (cm) | 24.04 ± 0.95 | 23.79 ± 0.72 | 21.39 ± 0.91 |
| EDL (mg)/tibia length (cm) | 5.53 ± 0.29 | 5.49 ± 0.15 | 5.24 ± 0.20 |
| **Phenotype** |  |  |  |
| Body mass (g)  before  after | 24.02 ± 0.51  24.90 ± 0.77 | 24.23 ± 0.49  25.22 ± 0.34 | 24.26 ± 0.65  24.85 ± 0.60 |
| Lean mass (g)  before  after | 20.76 ± 0.53  20.83 ± 0.52 | 20.67 ± 0.43  21.02 ± 0.44 | 21.12 ± 0.57  20.82 ± 0.41 |
| Fat mass (g)  before  after | 2.35 ± 0.12  3.12 ± 0.26 | 2.32 ± 0.15  2.87 ± 0.19 | 2.56 ± 0.21  2.86 ± 0.25 |
